# Shaping infants’ social brains through vicarious social learning: the importance of positive mother–father interactions

**DOI:** 10.3389/fpsyg.2024.1419159

**Published:** 2024-10-02

**Authors:** Sofie Rousseau, Nuphar Avital, Yuliya Tolpyhina

**Affiliations:** ^1^Paul Baerwald School of Social Work and Social Welfare, The Hebrew University of Jerusalem, Jerusalem, Israel; ^2^School of Education, Ariel University, Ariel, Israel

**Keywords:** infancy, early childhood, vicarious social learning, mother–father relationship, interparental positivity, neural development, EEG, social development

## Abstract

**Introduction:**

This study is the first to assess whether infants’ developing social brains may be susceptible to the vicarious social experience of interparental positivity. Specifically, we explored whether infants’ exposure to interparental positivity may vicariously shape their neural substrates of social development.

**Methods:**

In a sample of 45 infants (*M*_AgeMonths_ = 11.01; 48.9% girls), infant left-frontal resting alpha electroencephalogram (EEG) asymmetry was derived as a reliable indicator of neural substrates linked to adaptive social development. Moreover, positive characteristics of the mother–father couple relationship were assessed both by means of observation and self-report by mother and father. Importantly, various relevant covariates were considered, including interparental negativity (observed and self-reported), as well as infants’ direct caregiving experiences and duration of infant exposure to mother–father relationship-dynamics (parent-report).

**Results:**

Results indicated that higher levels of observed interparental positivity were associated with greater infant left-frontal alpha EEG asymmetry, even after accounting for covariates (β’s > 0.422).

**Discussion:**

The current study’s results are first to suggest that positive vicarious social experiences in infants’ day-to-day lives play a significant role for early neural development.

## Introduction

Abundant previous work has emphasized how neural foundations of social development are formed during early childhood, when the brain shows substantial structural and functional development (Bell and Fox, 1994; Knudsen, 2004; MacNeill et al., 2018; Tottenham and Sheridan, 2010). Specifically, previous work has highlighted the significant impact of various components of the direct interaction between infants and caregivers (for review see Ilyka et al., 2021). Yet, the importance of infants’ social experiences beyond their direct interactions with others currently remains largely unexplored. The goal of the present study is to examine the relevance of positive vicarious social experiences. Specifically, in line with modeling theories, which have received initial support in developmental neuroscience (Addabbo et al., 2020; Drew et al., 2018; Missana and Grossmann, 2015; Müller et al., 2017; Theall-Honey and Schmidt, 2006), we hypothesize that infants’ mere observations of high-quality interaction between mothers and fathers may vicariously shape their social neural development. Such understanding would carry critical implications for advancing our insight of early brain development. Moreover, it would hold crucial importance for early childhood developmental practice, highlighting the neurodevelopmental importance of fostering high-quality interaction between parents, and potentially also other caregiving figures, during the sensitive postpartum period.

### Mother–father relationship quality and infant social development

Decades of behavioral research and theory have indicated that high quality mother–father relationships are associated with various indices of young children’s adaptive social development (Balfour et al., 2018; Cummings and Davies, 2002; Don et al., 2024; Frosch and Mangelsdorf, 2022; Van Eldik et al., 2020). In this previous work, high quality mother–father relationships have been consistently characterized by parents’ reciprocal enjoyment, support and care. Specifically, during unstructured interaction, high quality mother–father relationships are observed through instances of engagement (e.g., initiating conversation, demonstrating involvement, displaying visual regard), mutual exchange of positive affect (e.g., smiling, positive vocalizations, affectionate touch), cooperation, joint focus, balanced relative contribution to the interaction, and reciprocal affirmation of one another’s contributions (Frosch and Mangelsdorf, 2022; Jessee et al., 2018). In addition, under conflict, high quality relationships are typified by cooperation as witnessed in calm discussion, constructive problem solving, affectionate and supportive behaviors, effective conflict resolution, and presenting opinions in a constructive and respectful manner (Davies et al., 2016; Easterbrooks et al., 1994; Frosch and Mangelsdorf, 2022; Jessee et al., 2018).

Indeed, abundant theoretical work has suggested that through the repeated exposure to high quality mother–father interactions, infants develop an understanding of how individuals can share experience, laying the foundation for more complex social understanding (Balfour et al., 2018; Cummings and Davies, 2002). Empirical data has indicated that interparental interactions that are high in the above reviewed positive behaviors, elicit real-time positive emotional responses in infants and children who observe them (Du Rocher Schudlich et al., 2011; McCoy et al., 2009). Regarding child sequelae that is associated with the repeated exposure to high quality mother–father interactions, meta-analytic work has indicated links with higher levels of infant and child overall adjustment (van Eldik et al., 2020), as well as higher levels of adaptive infant and child social behavior (Hosokawa and Katsura, 2017; McCoy et al., 2009; Neppl et al., 2019). Importantly, previous research as well as intervention work has successfully manipulated mother–father relationship quality (Cowan et al., 2011; Shapiro et al., 2015), leading to observed positive changes in children’s real-time emotional and social reactions as well as long-term development.

### Early development of the “social brain” as indexed by left frontal resting alpha EEG asymmetry

Independent of the research lines elaborated above, a large body of studies has reliably shown how specific neural substrates are underlying social development in young children (Bowman et al., 2019; Rice and Redcay, 2015). In older children and adults, functional magnetic resonance imaging (fMRI) evinces robust findings of a network of brain regions engaged for social processing, including the medial prefrontal cortex, temporoparietal junction, inferior frontal gyri, precuneus, superior temporal sulcus, superior temporal gyrus, insula, and amygdala (Fallon et al., 2020; Schurz et al., 2021). These conclusive results expose a “social neural network” or “social brain.” In early childhood, both MRI and electrophysiology (EEG) studies have confirmed that individual variances in maturation and organization of this “social brain,” as measured at rest when a participant is not given any specific cognitive task to complete, are related to individual differences in adaptive socio-emotional performance, including joint attention, empathy, and theory of mind (Bowman et al., 2019; Rice and Redcay, 2015). EEG is particularly invaluable for examining neural development throughout the first years of life, due to its advantages over fMRI in quiet measurement, quick application, and not requiring separation between young children and caregivers.

One important EEG signature previously related to emerging adaptive behavior comprises resting frontal alpha asymmetry, i.e., the subtraction of resting EEG activity in corresponding left and right hemisphere electrode pairs. In normative child and adult populations, research has consistently identified left frontal resting alpha EEG asymmetry as a reliable marker of adaptive regulatory behaviors, increased positive affect during social interactions, as well as greater tendency for social initiation (Fox et al., 1995). Conversely, lower levels of this marker, characterized by right frontal asymmetry, are related to maladaptive regulation (Garrison et al., 2022) and social withdrawal (Smith and Bell, 2010). In line with these above findings, several EEG studies involving infants and early childhood samples have revealed positive associations between left-frontal resting alpha asymmetry and adaptive socio-emotional outcomes, including infants’ empathic prosociality (Frenkel et al., 2024), emerging toddlers’ joint attention skills (Mundy et al., 2000), emerging toddlers’ empathic and prosocial behaviors (Paulus et al., 2013), and young children’s social capacities in peer-interactions (Fox et al., 1995).

These established associations between the EEG signature of increased left-frontal resting alpha asymmetry and emerging socio-emotional capacities confirm the presence of a “social brain” that can be indexed with EEG from early infancy onwards (Missana and Grossmann, 2015; Mundy et al., 2000; Paulus et al., 2013). This social EEG signature can then be used to detect changes in neural activity as a function of early caregiving experiences.

### Early caregiving experiences and infant left frontal resting alpha EEG asymmetry

Various previous studies have proposed that experiences central to infants’ brain development are embedded in the early caregiving context (for a review see Ilyka et al., 2021). Yet, these studies have focused on assessing early caregiving practices aimed directly at the young child. For example, past work has clearly demonstrated that normative variations in infants’ and children’s direct experiences with positive, warm and responsive caregiving behavior within the interactions with their caregivers, are associated with particular patterns of EEG activity, including frontal alpha EEG asymmetry (Frenkel et al., 2024; Hane and Fox, 2006; Hane et al., 2010; Ilyka et al., 2021; Orekhova et al., 2006; St John et al., 2016).

Intriguingly, throughout the past decades, human neuroscience has evinced remarkable similarity between individuals’ neural activity while actively and directly interacting with others and individuals’ neural activity while passively observing others experiencing alike interactions (Morelli et al., 2015; Murray et al., 2012; Peled-Avron and Woolley, 2021). Notably, a handful of these studies have included infants and children (Addabbo et al., 2020; Drew et al., 2018; Missana and Grossmann, 2015; Müller et al., 2017; Theall-Honey and Schmidt, 2006). Although this previous work has greatly contributed to our current knowledge, it has mainly focused on examining the link between positive vicarious caregiving experiences as temporarily manipulated in the lab, and the subsequent real-time activation of infants’ social brains. As such, it currently remains unknown whether individual differences in young children’s continued and repeated exposure to positive vicarious caregiving experiences, encountered daily within their caregiving environments, may foster the maturation and organization of their “social brain” as measured during resting-state tasks.

Some bodies of work have started to address the importance of continued and repeated exposure to vicarious caregiving experiences for young children’s social brain development. For example, previous studies suggest that infants who witness intimate partner violence between their mothers and fathers during the first five years of life, experience lasting effects on their brain development (for review see Mueller and Tronick, 2019; Tsavoussis et al., 2014). Additional research has highlighted the role of neighborhood socio-economic (dis)advantage as a significant source of ‘vicarious social experience’ influencing early brain development (Gard et al., 2021; Hyde et al., 2022). Overall, this previous research on children’s vicarious social experiences has mainly focused on negative aspects, indicating that neighborhood socio-economic disadvantage as well as negative mother–father interactions are associated with children’s neural developmental disadvantage and that the absence of such negative vicarious social experiences is crucial for preventing neural developmental disadvantage. Moreover, the presence of neighborhood socio-economic advantage seems important for building prosperous neural development. However, whether positive vicarious experiences of observing positive mother–father interactions may contribute to thriving neural development, even while accounting for other factors such direct positive parent–child relationships or interparental negativity, remains currently unknown.

Acknowledging the importance of such positive vicarious caregiving experiences not only for real-time temporary neural functioning but also for the maturation and organization of neural social substrates would greatly advance our understanding of the developing social brain. Moreover, it would hold crucial practical importance, providing a solid foundation for fostering the quality of daily interparental interactions during the postpartum period.

### The current study

The current study is first to assess whether positive mother–father relationship quality is associated with infant “social brain” development, as measured by left frontal alpha EEG asymmetry at rest. Specifically, in line with previous research on the importance of positive direct caregiver-infant interaction for infant neural development (Ilyka et al., 2021), as well as vicarious neuroscience suggesting alike effects for temporary indirect vicarious caregiving experiences (Addabbo et al., 2020; Drew et al., 2018; Marshall et al., 2013; Missana and Grossmann, 2015; Müller et al., 2017; Theall-Honey and Schmidt, 2006), we hypothesize that young infants’ continued and repeated daily exposure to high quality positive mother–father interactions would be associated with higher levels of left frontal resting alpha EEG asymmetry. Importantly, in order to elucidate the unique contribution of positive mother–father relationship quality, key covariates such as interparental negativity, infants’ direct experiences with positive, warm and responsive caregiving behavior (Ilyka et al., 2021; Van Eldik et al., 2020), and duration of infants’ daily exposure to mother–father relationship dynamics, will be controlled for.

## Methods

### Participants

A total of 47 families participated in this study. To be eligible, mother and father (i.e., legal guardians) had to be living together and have a 12-month-old infant. Additionally, they had to be willing to participate in a one-hour home visit with the mother, father, and infant present, as well as to independently fill out a set of online questionnaires at the end of the home visit. Exclusion criteria were non-proficiency in Hebrew, twin infants, and serious infant medical issues or developmental delays (e.g., born before 37 weeks; birth weight < 2.5 kg; diagnosed neurological conditions like epilepsy or cerebral palsy; genetic disorders such as Down syndrome; severe birth complications like hypoxia; significant central nervous system infections like meningitis; metabolic disorders like phenylketonuria; visual or hearing impairments; medication affecting brain activity; strong family history of neurodevelopmental disorders; significant medical conditions like congenital heart defects; diagnosed developmental delays). Furthermore, participation was limited to families residing within a maximum 90-min drive from the central region of Israel.

From the 47 families that finished data-collection, 2 were excluded for analyses, because throughout the study infants showed signs of developmental delays. As such, the final sample considered for analyses included 45 families. For 6 of these families, there were serious overall interferences during EEG data collection, and as such, infant EEG data was missing. Interferences included infant cap refusal, infant tiredness, infant fussiness, interferences of the parents, and interferences of the family dog. For one additional family, parents did not fill out the questionnaires even after various friendly reminders of the research team and as such, the questionnaire data was missing. Two other families had missing data for the observed mother–father interactions due to issues with low sound quality and camera angles, which prevented behavioral coding of the interactions. Missing data was addressed in a statistical manner (see below). Independent sample t-tests and χ2 tests indicated that the two families that were excluded due to child developmental delays did not significantly differ from included families for age child, sex child, age mother, age father, years of education mother, years of education father, religion mother, religion father, religiosity mother, religiosity father, number of children, and household income (*p*’s > 0.447). Moreover, there were no significant differences in these variables between families with missing data and families without missing data (*p*’s > 0.199). Table 1 provides detailed demographic characteristics for the final study sample of 45 families.

**Table 1:** Demographic characteristics for the final study sample (*n* = 45).

Continuous variables	Min	Max	*M*	*SD*
Age child (months)	11.01	14.32	12.12	0.86
Number of children	1.00	9.00	2.07	1.47
Age mother (years)	24.19	44.69	33.84	5.26
Age father (years)	23.99	48.96	36.16	6.69
Years of education mother	11.50	25.00	16.30	3.19
Years of education father	12.00	24.00	14.94	2.95
**Categorical variables**	**Percentage**			
Married	100.0			
Sex child				
Female	48.9			
Male	51.1			
Religion mother				
Jewish	100.0			
Non-Jewish	0.0			
Religion father				
Jewish	97.7			
Non-Jewish	2.3			
Religiousness mother				
Secular	52.3			
Traditional	15.9			
Religious	25.0			
Very religious	6.8			
Religiousness father				
Secular	59.1			
Traditional	11.4			
Religious	22.7			
Very religious	6.8			
Monthly household income*				
Lower than $3,000	18.2			
Between $3,000 and $4,500	27.3			
Above $4,500	54.5			

*Data indicates that the majority of participants had a monthly household income in line with or exceeding Israel’s average monthly household income at the time of recruitment, which was approximately $4,500 according to the Israel Central Bureau of Statistics (2019).

### Procedure

All methods were carried out in accordance with relevant guidelines and regulations. All research protocols were approved by the first author’s institutional review board (approval number: AU-SOC-SR-20230125; approval date: 22.1.2023). Participants were recruited through social media channels and WhatsApp groups, particularly those catering to new mothers and fathers. Parents who expressed interest were contacted by the lead research assistant to assess compatibility, and suitable families were then scheduled for a home visit. Given the great sensitivity of EEG data to age, a concerted effort was made to schedule all visits at exactly two weeks before the infant’s first birthday. However, several families encountered logistical challenges that required scheduling the visit slightly before or after that specific date. Moreover, some planned visits underwent last-minute rescheduling due to infant illness or unforeseen family events. Data was collected between February 10th 2023 and June 30th 2023.

The home visit was carried out by a team of three research assistants. The lead research assistant took charge of all primary interactions with the family and infant, a second research assistant handled EEG measurements and on-site data quality control, while the third research assistant provided support where needed. Upon arriving at the family’s home, after initial greetings, both mother and father independently signed an informed consent form. Parents provided consent for both their own participation and that of their infant. Subsequently, parents independently completed a questionnaire identifying topics on which they disagreed. This was followed by a 5-min discussion between mother and father on the topic with the most disagreement. Afterwards, the infant was seated on the mother’s lap, and the EEG net was placed on the infant’s head. Two minutes of continuous EEG were recorded while the infant watched machine-blown bubbles. During EEG data-collection, the father stood behind the mother and the infant, out of sight, and at a distance of more than two meters. Fathers were asked to remain silent and refrain from any actions that could draw the infant’s attention. Mothers were instructed to hold the infant on their lap without engaging in other activities, including movements, speaking, or caressing the infant. Afterwards, parents independently completed online questionnaires created using Google Forms software. Questionnaires assessed positive and negative characteristics of the mother–father relationship, demographic information, warm and responsive direct caregiving behavior, and the duration of infant exposure to mother–father relationship dynamics. Parents were encouraged to complete the questionnaires at the end of the home visit, while the research team attended to the infant. For those who preferred to do so later, we requested completion within one week after the home visit. Each family received a gift coupon of 100 NIS ($30) as compensation for participation.

### Measures

#### Positive and negative characteristics of the mother–father relationship

Interparental positivity and negativity were assessed by means of self-report and observation. Concerning self-report, mothers and fathers both independently completed the Revised Dyadic Adjustment Scale (RDAS; Busby et al., 1995). The RDAS is the widely used short form of the original Dyadic Adjustment Scale (Spanier, 1976), consisting of 14 self-report items. In line with the current study’s research hypotheses, the subscales dissatisfaction (i.e., 5 negatively loading items of the satisfaction scale, e.g., How often do you and your partner “get on each other’s nerves?”; How often do you or your partner leave the house after a fight?), satisfaction (i.e., 4 positively loading items of the satisfaction scale, e.g., How often do you confide in your partner?; How often do you kiss your partner?; In general, how often do you think that things between you and your partner are going well?), and cohesion (5 items, e.g., How many of your activities outside the house do you and your partner do together? At what frequency do you and your partner exchange ideas? How often do you and your partner laugh together?) were considered. The subscales satisfaction and cohesion represent positive characteristics of mother–father interaction whereas the subscale dissatisfaction represents interparental negativity. Following standard practice, items were scored on different Likert-type scales, i.e., ranging from 0 (never) to 4 (each day), e.g., “How often do you kiss your partner?”; ranging from 0 (not at all) to 4 (all of them), e.g., “How many of your activities outside the house do you and your partner do together”; ranging from 0 (never) to 5 (always), e.g., “At what frequency do you and your partner exchange ideas?”; ranging from 0 (not happy) to 6 (happy), e.g., “Rate the level of happiness that you experience in your relationship.” Total scores for each of the three subscales were calculated separately for mothers and fathers by averaging their item scores, which is in line with original scoring instructions. Previous research has identified strong psychometric properties for the RDAS (Busby et al., 1995). In the current study, Cronbach’s alphas were good for the satisfaction scale (0.73 for fathers, 0.70 for mothers), dissatisfaction scale (0.63 for fathers, 0.71 for mothers), and cohesion scale (0.69 for fathers, 0.77 for mothers). Significant correlations were seen between mother and father report for all three scales: satisfaction scale (*r* = 0.55; *p* < 0.001), dissatisfaction scale (*r* = 0.39; *p* = 0.011), and cohesion scale (*r* = 0.30; *p* = 0.050). As such, to obtain the most valid and reliable measure of the daily dynamics in the mother–father relationship as witnessed by the infant, and in line with scoring guidelines, mother and father scores were combined for each subscale, by averaging.

Regarding observation, positive and negative characteristics of the mother–father relationship were assessed during a discussion task. In line with similar procedures in previous research (Davies et al., 2016; Du Rocher Schudlich et al., 2011) parents first independently completed a disagreement questionnaire. Specifically, for a total of 23 topics (e.g., One of the partners promises to do something and does not do it; Disagreeing on how to deal with the children), parents filled out whether the topic was discussed during the last month, and if yes, how many times as well as how heated the discussion was on a Likert-type scale ranging from 1 (calm) to 5 (angry). Subsequently, for each topic that was discussed during the last month, disagreement scores were calculated for mother-report and father-report separately, by multiplying the number of times it was discussed by the level of heatedness. Then, disagreement scores were ranked separately for both mother and father, and the topic on which they both disagreed the most was selected for discussion. Specifically, they were asked to discuss the topic for 5 minutes, trying to reach a solution. If they finished before the allotted time, they could request the next topic from the research assistant. The child was present during the discussion and parents were instructed to respond to their child and interact with their child as they normally would at home during comparable interparental discussions. Interactions were behaviorally coded offline from video recordings, by means of the coding scheme for interparental interactions (Frosch and Mangelsdorf, 2022). More specifically, the 5-minutes interactions were macro-coded for 11 dimensions: engagement, enjoyment, expression of positive affect mother, expression positive affect father, cooperation, balance/reciprocity, sensitivity/support, conflict resolution/satisfaction, irritation, expression of negative affect mother, expression of negative affect father. All scales were coded on a 7-point Likert-type scale (1 = low, 7 = high) by a main experienced coder, who was blind to the other study data. A second experienced blind coder separately rated 13 videos (i.e., 30% of the sample). Positive dimensions (engagement, enjoyment, expression of positive affect mother, expression positive affect father, cooperation, balance/reciprocity, sensitivity/support, conflict resolution/satisfaction) were averaged to create a total positive mother–father interaction score, whereas negative dimensions (irritation, expression of negative affect mother, expression of negative affect father) were averaged to create a total negative mother–father interaction score. Single measures intraclass reliability coefficients (ICC) were satisfactory for all scales, ranging between 0.61 and 0.94, with an overall average ICC score of 0.74 for the total positive mother–father interaction score and an overall average ICC score of 0.80 for the total negative mother–father interaction score.

#### EEG data-collection and processing

EEG data was recorded while infants watched 2 minutes of machine blown bubbles, thereby tapping into the ‘resting’ or ‘idling’ brain, recorded when a participant is not given any specific cognitive task to complete. EEG data was recorded continuously from scalp electrodes using Ant Neuro’s (Ant Neuro, Netherlands) 64-channel net (**wave**guard^™^ capt; Equidistant hexagonal electrode layout - ANT/Duke layout); **eego**^™^ EEG amplifier (64ch + 24 ch, 16 kHz), and **eego**^™^ mylab recording software. Electrical impedances were kept below 50 kΩ, and Cz was used as the recording reference. Signals were amplified with a 0.1 Hz to 100 Hz elliptical bandpass filter and digitized at a 500 Hz sampling rate.

The EEG recording setup was filmed and processed offline, with the lead research assistant identifying segments of behavioral interference and artifacts. Specifically, following segments were identified: instances where infants initiated interaction with their mothers, periods of pronounced infant fussiness, moments where infants averted their gaze from the bubbles, and interruptions from others (e.g., parents making comments although instructed not to interfere). To prevent excessive fragmentation of the EEG recording, only interferences lasting two seconds or more were identified. Video recordings were synchronized with each participant’s EEG recording, and the onset and end of behavioral interferences were marked. These marked segments were subsequently excluded from EEG data analyses.

EEG data was analyzed offline using the interactive MATLAB toolbox EEGLAB (MATLAB version R2023b; EEGLAB version 2023). Several key steps were undertaken. First, the data was re-referenced to an average reference configuration. Subsequently, artifact identification and removal occurred in three steps. First, we applied a bandpass filter with a lower cutoff frequency of 1 Hz an upper cutoff frequency of 40 Hz. Second, Independent Component Analysis with Infomax Algorithm was applied to separate the neural signal from interfering electrical signals in the EEG trace. Per default, components with more than 90% probability of stemming from eye artifacts (eye blinks and eye movements) or other muscle movement, were removed. Third, within one second segments, we rejected channels with a maximum voltage fluctuation exceeding 150 μV or a voltage discrepancy compared to adjacent channels exceeding 30 μV. Time segments with more than 35% of such problematic channels were excluded from further analyses. Next, artifact corrected EEG files were analyzed with MATLAB script code based on Welch’s method, calculating average power at frequencies of 6, 7, 8, and 9 Hz for each electrode channel.

**Figure 1:**
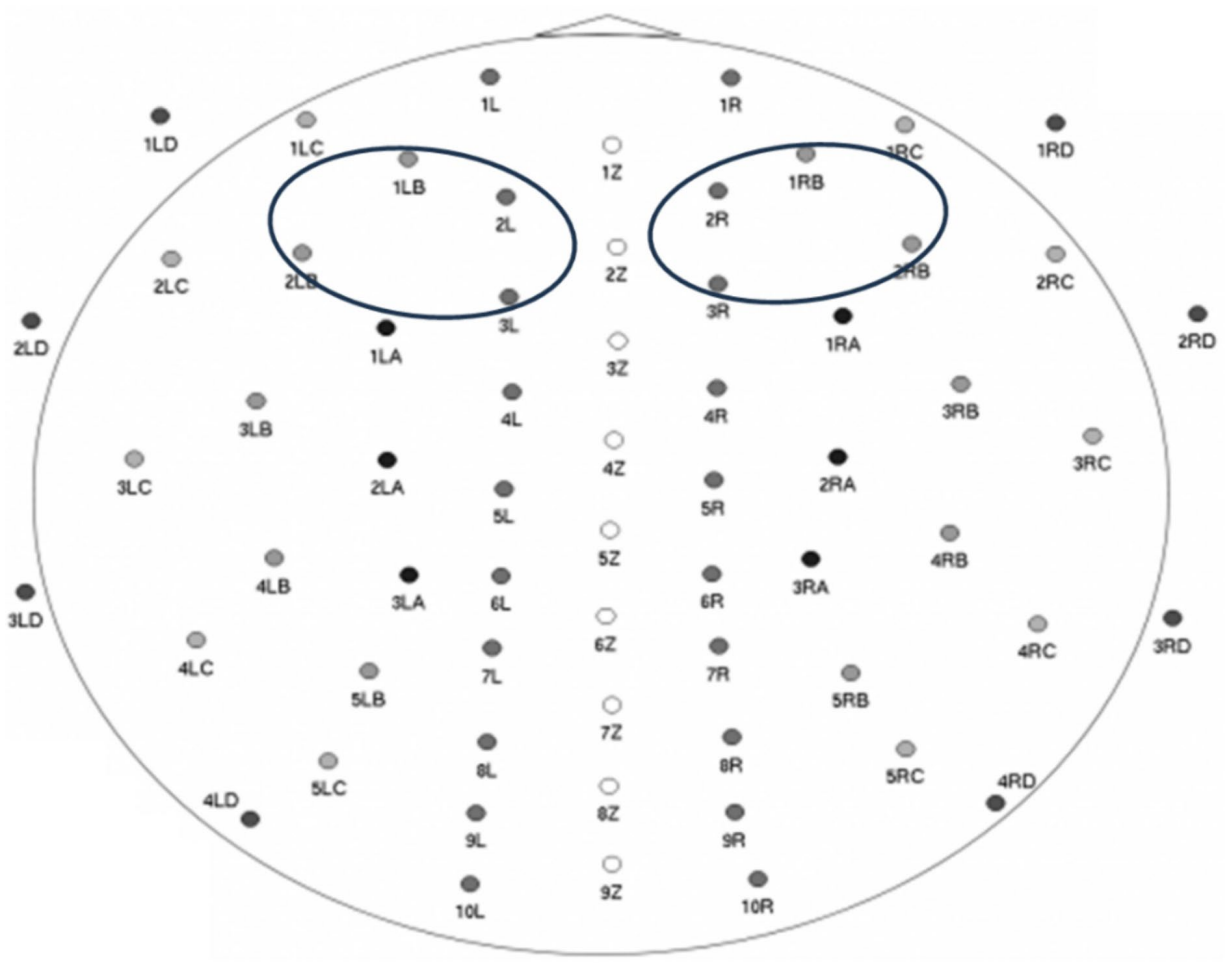
**wave**guard^™^ cap; 64-channel map + electrodes used for calculating infant frontal alpha EEG asymmetry (image source: ANT Neuro BV; used with permission).

Subsequently, power was averaged within two regions (see Figure 1): a cluster of 4 right frontal electrodes providing a high-density representation of F4 locations (electrode #s 2R; 3R; 1RB; 2RB), and their homologous left frontal electrodes providing a high-density representation of F3 locations (electrode #s 2 L; 3 L; 1LB; 2LB). Infant’s frontal EEG alpha asymmetry was then calculated following standard practice, by subtracting the natural log-transformed left regional mean from the natural log-transformed right regional mean. Higher asymmetry scores indicate higher levels of relative left frontal alpha asymmetry (and lower levels of relative right frontal alpha asymmetry), whereas lower asymmetry scores indicate lower levels of relative left frontal alpha asymmetry (and higher levels of relative right frontal alpha asymmetry). On average, infants had 2.27 min of usable EEG data (*SD* = 0.31; minimum 1.78 min; maximum 3.05 min). Importantly, data quantity and infant age were controlled for by regressing these variables on the EEG asymmetry measure and using the saved standardized residuals in all analyses.

#### Control variables

In addition to controlling for negative characteristics of the mother–father relationship, whose measurement is detailed above, several other control variables were assessed through maternal and paternal reports. Specifically, demographic variables including infant sex, number of children in the household, mother’s age, and father’s age were considered. Moreover, we controlled for maternal and paternal warm and responsive direct caregiving behaviors due to previous research indicating their associations with infant frontal alpha EEG asymmetry (Ilyka et al., 2021) and with mother–father relationship quality (Van Eldik et al., 2020). Finally, the relevance of the duration of infant exposure to mother–father relationship dynamics was considered. In what follows, we elaborate on the measures used to assess these two latter control variables.

##### Maternal and paternal warm and responsive direct caregiving behavior

Mothers and fathers each independently completed the “Warmth” subscale from the Ghent Parental Behavior Scale for Toddlers (GPBS; Van Leeuwen et al., 2011). A total of 15 items (e.g., I play with my child; When my child cries, I check why s(he) is crying; I imitate the sounds that my child makes) were answered on a Likert-type scale ranging from 1 (never) to 5 (always). In line with previous research, all item scores were averaged to create a total score, for which higher levels indicated greater warm and responsive caregiving behavior (Van Keer et al., 2017; Van Leeuwen et al., 2011). Previous research has supported the strong reliability and validity of the scale for measuring parental caregiving warmth and responsiveness (Van Keer et al., 2017; Van Leeuwen et al., 2011). In the current study, Cronbach’s Alpha was 0.90 for father report and 0.76 for mother report.

##### Duration of infant exposure to mother–father relationship dynamics

Duration of infant exposure to mother–father relationship dynamics was assessed by means of two questions answered by both mother and father: “What is the average amount of hours that the three of you (mother, father, and infant participating in this study) spend together each day (with or without other children or adults)?”; “During the last four weeks, what was the average amount of hours that your child watched you and your partner discuss any topic?” For each question, mother and father reports were averaged and these average scores were used in analyses.

### Data-analytic approach

All data was analyzed by means of the statistical software SPSS, version 24, including the add-on PROCESS macro for conditional analyses (Hayes, 2017). Little’s MCAR test indicated that missing data was missing completely at random (χ2 (101) = 92.53, *p* = 0.714), and as such, multiple imputation was used to reliably replace missing values (Schafer and Graham, 2002; Sterne et al., 2009). The imputation model contained all main study variables, as well as all control variables (negative characteristics of the mother–father relationship; Infant sex; Number of children in the household; Age mother; Age father; Maternal warm and responsive caregiving behavior; Paternal warm and responsive caregiving behavior; Duration of infant exposure to mother–father relationship dynamics) which were added as auxiliaries. A total of ten completed datasets were created. Given a missing value rate below 50%, the accuracy of statistical estimates based on ten imputations is higher than 95% (Schafer and Graham, 2002). Importantly, multiple imputation can be employed without risk of power loss, and thus statistical power can be calculated based on the actual sample size, including participants with missing data (Graham et al., 2007). For all statistical analyses, pooled results from the ten imputed datasets were reported. Bonferroni correction was not applied, as to not increase the risk of Type II errors, given the sample size in the current study (Nakagawa, 2004; Perneger, 1998).

Statistical analyses commenced by calculating correlations and descriptive statistics for all study variables, including main and control variables. Subsequently, main analyses were conducted. First, a series of simple regression analyses was run, assessing associations between the various indicators of interparental positivity (observed characteristics of positive mother–father interactions, self-reported satisfaction, self-reported cohesion) and infant frontal resting alpha EEG asymmetry. Second, for each indicator of interparental positivity that showed a significant effect in this first round of analyses, a series of multiple regression analyses was performed to determine whether the effect remained significant when accounting for control variables (negative characteristics of the mother–father relationship; Maternal warm and responsive caregiving behavior; Paternal warm and responsive caregiving behavior; child sex; number of children in the household; age mother; age father). In order not to bloat the statistical models, a separate multiple regression analysis was run for each control variable. Specifically, control variables were added to the first step of the regression analysis, while the indicator of interparental positivity was added to the second step. As such, a significant F-change statistic for the second step of the model indicated a significant association between the indicator of interparental positivity and infant EEG, above and beyond the effect of the control variable. Finally, for each indicator of interparental positivity that showed a significant effect in the first round of analyses, two moderation analyses were run to assess whether the association between this indicator of mother–father relationship quality and infant EEG was moderated by duration of infant exposure to mother–father interaction (first moderation analysis) and by duration of infant exposure to mother–father discussion (second moderation analysis).

Power requirements were assessed in the program G*Power (Faul et al., 2007). Given the overall scarcity of previous research on the link between the couple relationship quality and infant neural development or functioning, conservative effect sizes of f^2^ = 0.2 were hypothesized. Power analyses indicated that a sample size of 42 was necessary to assure 0.80 power for the F change test in the multiple regression analyses as well as for the interaction coefficient in the moderation analyses, at an alpha level of 0.05. Our analyses represent a confirmatory effort in that specific hypotheses were tested, yet the study was not preregistered.

## Results

Outliers were examined for all variables. Except for one child’s EEG measurement, which was 4 SD below the mean, no values exceeded 2 SD. Given the disagreement in the current literature about whether or not to exclude children with outlying values (Bakker and Wicherts, 2014), all analyses were conducted both including and excluding this family. The results remained consistent regardless. For reasons of parsimony, in what follows, only the analyses excluding this family are presented.

Table 2 presents descriptive values and correlations for all study variables, including main and control variables.

**Table 2:** Descriptive values and correlations for all study variables.

		Descriptive			Correlations															
		*n*	*M*	*SD*	1.	2.	3.	4.	5.	6.	7.	8.	9.	10.	11.	12.	13.	14.	15.	16.
1.	MFRQ: observed—positive	44	4.40	1.01		−0.64^a^	−0.08	−0.05	−0.16	0.44^c^	−0.17	0.09	0.09	−0.27	−0.35^c^	−0.16	−0.04	0.05	0.14	0.23
2.	MFRQ: observed–negative	44	2.15	1.18			−0.03	0.07	−0.11	−0.10	0.10	−0.12	−0.18	0.30	0.24	−0.11	0.11	−0.13	−0.07	−0.16
3.	MFRQ: self−report–satisfaction	44	5.26	0.62				−0.67^c^	0.24	0.05	0.00	−0.05	−0.01	0.29	0.32	0.03	−0.25	0.12	0.03	−0.16
4.	MFRQ: self−report–dissatisfaction	44	1.86	0.46					−0.13	−0.12	0.22	0.03	0.01	−0.26	−0.20	−0.02	0.19	−0.03	0.12	0.17
5.	MFRQ: self−report–cohesion	44	4.04	0.60						−0.22	0.07	0.01	0.13	0.42	0.36	0.14	0.11	−0.19	0.03	−0.09
6.	Infant frontal resting alpha EEG asymmetry	44	0.15	0.90							−0.22	−0.15	0.12	−0.09	−0.22	−0.06	0.02	0.10	0.05	0.07
7.	Minutes of usable EEG	44	98.69	47.04								−0.04	−0.17	0.04	0.20	−0.05	−0.18	−0.05	−0.02	0.01
8.	Warm and responsive direct caregiving behavior mother	44	4.30	0.38									−0.19	−0.03	−0.24	−0.21	0.51	−0.05	−0.09	0.10
9.	Warm and responsive direct caregiving behavior father	44	4.06	0.68										−0.31	−0.13	0.07	0.17	−0.13	−0.04	−0.10
10.	Time infant spends with both parents	44	1.98	0.65											0.53^b^	0.15	−0.13	−0.03	−0.11	−0.15
11.	Time infant observes discussions between parents	44	1.78	0.57												0.14	−0.29	−0.12	−0.13	−0.17
12.	Infant age in months	44	12.11	0.87													−0.10	0.06	0.02	−0.11
13.	Infant sex^I^	44	0.50	0.51														−0.19	0.05	0.08
14.	Number of children in the household	44	2.37	1.74															0.25	0.26
15.	Age mother in years	44	35.96	6.64																0.81^a^
16.	Age father in years	44	33.73	5.37																

MFRQ = mother–father relationship quality; ^I^dummy coded: male = 1, female = 0; ^a^*p* < 0.001; ^b^*p* < 0.01; ^c^*p* < 0.05.

**Figure 2:**
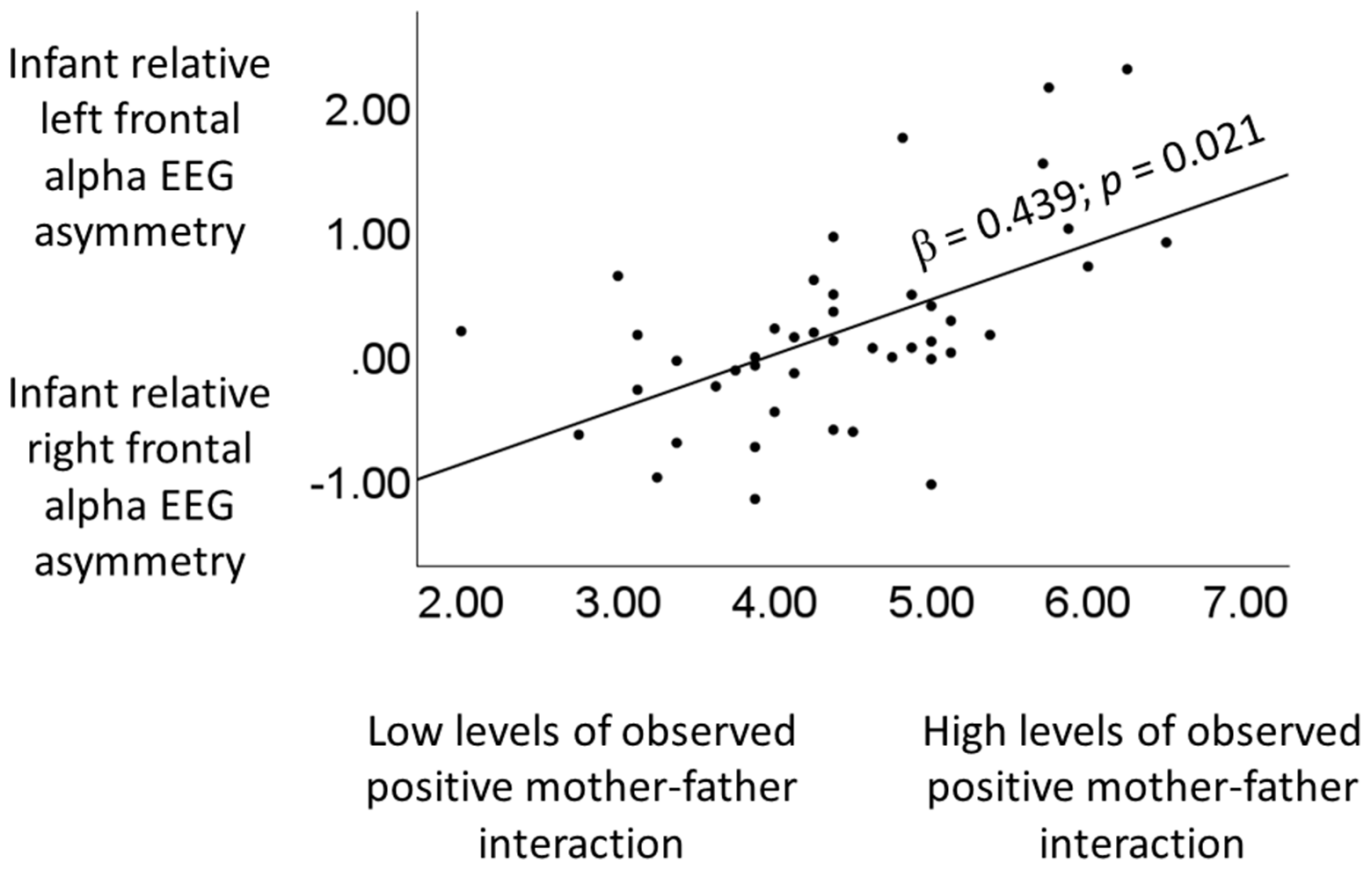
Higher levels of observed positive mother–father interaction are associated with higher levels of infant relative left frontal alpha EEG asymmetry.

A series of simple regression analyses assessing the association between the various indicators of interparental positivity (observed characteristics of positive mother–father interactions, self-reported satisfaction, self-reported cohesion) and infant relative left frontal resting alpha EEG asymmetry, indicated that higher levels of observed characteristics of positive mother–father interactions predicted higher relative left frontal resting alpha EEG asymmetry (β = 0.439; *SE* = 0.165; *p* = 0.021; *R^2^* = 0.197; see Figure 2). There were no significant results for the self-reported indices of interparental positivity, i.e., satisfaction and cohesion (*p*’s > 0.332; *SE*’s < 0.491). Next, a series of multiple regression analyses was run, to test whether the significant result for observed characteristics of positive mother–father interactions held above and beyond various covariates. Specifically, we ran separate models for each covariate (observed characteristics of negative mother–father interactions; self-reported dissatisfaction; Maternal warm and responsive caregiving behavior; Paternal warm and responsive caregiving behavior; child sex; number of children in the household; age mother; age father). In each model, the covariate was entered as an independent variable in the first step, and the variable “observed positive mother–father relationship characteristics” was entered in the second step. In all models, observed positive mother–father relationship characteristics remained a consistently strong and significant predictor of infant left frontal alpha EEG asymmetry (β’s > 0.422; *SE*’s < 0.176; *p*’s < 0.028; *R*^2’^s > 0.188). Indeed, in all multiple regression models, significant F Change statistics were seen for the second step of the model, in which mother–father relationship quality was added (*F* change statistics (41, 1) > 10.245; *p*’s < 0.011). None of the covariates were significant unique predictors of EEG asymmetry when assessed together with observed positive mother–father relationship characteristics in the second step of the models (*p*’s > 0.203; SE’s < 0.536).

Finally, moderation analyses indicated that the association between observed characteristics of positive mother–father interactions and infant EEG asymmetry was not significantly moderated by neither the time that the infant spends with both parents (β = 0.025; *SE* = 0.229; *p* = 0.332), nor by the time that the infant observes discussions between both parents (β = 0.037; *SE* = 0.225; *p* = 0.337).

## Discussion

Previous research has indicated that the neural foundations of adaptive socio-emotional development predominantly form within the first years of life (Bell and Fox, 1994; Knudsen, 2004; MacNeill et al., 2018; Tottenham and Sheridan, 2010). Building on this understanding, the past decades have significantly contributed to unraveling early malleable factors that may foster or hinder the development of neural substrates underlying socio-emotional capacities. The caregiving environment, and particularly children’s direct caregiving experiences such as maltreatment (Almas et al., 2012; Marshall et al., 2008), normative variations in maladaptive caregiving practices (Atzaba-Poria et al., 2017; Dawson et al., 1999), and normative variations in nurturing caregiving practices (Frenkel et al., 2024; Hane and Fox, 2006; Hane et al., 2010; Ilyka et al., 2021; Orekhova et al., 2006; St John et al., 2016), have been a focal point of research, with results emphasizing the crucial role of these factors for early neural development.

The current study builds on this past work and is the first to explore the neurodevelopmental importance of infants’ positive social experiences beyond their direct interactions with others. Specifically, drawing on vicarious learning theories, which have been supported in behavioral developmental science and partially supported in neurodevelopmental science (Addabbo et al., 2020; Drew et al., 2018; Missana and Grossmann, 2015; Du Rocher Schudlich et al., 2011; Hosokawa and Katsura, 2017; McCoy et al., 2009; Müller et al., 2017; Neppl et al., 2019; Theall-Honey and Schmidt, 2006; Van Eldik et al., 2020), we proposed the hypothesis that infants’ positive vicarious social experiences, as operationalized by their mere observation of interparental positivity, contribute to the development of their neural foundations of social development.

Study results indicated that, in line with initial hypotheses, greater observed positive mother–father relationship characteristics were significantly associated with greater infant left frontal resting alpha EEG asymmetry. Previous EEG research has consistently identified left frontal resting alpha EEG asymmetry as a reliable marker of key aspects of adaptive socio-emotional development, such as more advantageous regulatory capacity, higher levels of positive affect during social interactions, and greater tendency for social initiation (Fox et al., 1995). Conversely, previous research has shown that lower levels of this marker, characterized by right frontal asymmetry, are associated with maladaptive regulatory strategies (Garrison et al., 2022) and social withdrawal (Smith and Bell, 2010). Therefore, the results of the current study suggest that infants’ positive vicarious social experiences contribute to the development of their neural foundations underlying adaptive social skills and that under the absence of such high quality vicarious social experiences, the development of such advantageous neural substrate is at risk. Importantly, results were not moderated by duration of infants’ vicarious social experiences and as such they are likely to apply to all infants, including those who spend less time in the company of both caregivers.

Prior research has highlighted the significance of negative vicarious experiences of interparental violence and abuse for early brain development (see Mueller and Tronick, 2019; Tsavoussis et al., 2014). The relevance of performing additional research on the importance of positive vicarious experiences is supported by recent theoretical advancements, such as the Interparental Positivity Spillover Theory (IPST; Don et al., 2024). The IPST underscores the unique importance of interparental positivity for child development, emphasizing that its impact extends beyond merely the absence of interparental negativity. Particularly, negative interparental interactions, such as those characterized by aggression or contempt, undermine children’s sense of safety, leading to adverse emotional and social outcomes (Cummings and Miller-Graff, 2015; Davies and Martin, 2014; van Eldik et al., 2020). Yet, the mere absence of negative interparental interactions does not suffice to promote healthy social development, and particularly interparental positivity is believed to foster children’s positive emotions; positive perceptions of parents; and social behaviors through social learning (Ryan, 2017; Bowlby, 1988; Feeney and Thrush, 2010). Following a similar line of thought, one may hypothesize that while infants’ neural substrates for maladaptive social development might be shaped by their passive observation of maladaptive social interactions, merely lacking such observation may not necessarily foster the development of adaptive neural substrates. Instead, passive observation of adaptive social interactions may be crucial for promoting such development.

The current study assessed this premise by examining whether interparental positivity was uniquely associated with infant neural development and not merely explained by the absence of interparental negativity. Results indicated the unique importance of interparental positivity. Interestingly, however, indices of interparental negativity (e.g., observed characteristics of negative mother–father interactions; self-reported dissatisfaction) were not significantly linked to infant frontal resting alpha EEG asymmetry. One might argue that these results are contradictory to previous work, which indicates that children’s witnessing of intimate violence between mothers and fathers during early childhood has lasting effects on brain development (for review see Mueller and Tronick, 2019; Tsavoussis et al., 2014). However, in contrast to previous work, our study included a normative sample, and the measures of interparental negativity were designed to capture normative variations rather than extreme indices such as abuse and violence. As such, the results may indicate that exposure to normative variations in interparental negativity may be less influential for early brain development, compared to the significant impact of witnessing abuse and violence. Future research may further explore this premise, by including measures of more extreme characteristics of interparental negativity. Associated, future research might include different indices of normative variations in interparental negativity, such as difference scores between maternal and paternal reports.

The current study’s results entail important practical significance, especially when considering the heightened vulnerability of the couple relationship during the postpartum period. Indeed, various theoretical, clinical, and empirical writings have described the arrival of a new infant as a family crisis, leaving all members in a state of confusion and uncertainty (Shapiro et al., 2015). While prior behavioral research has indicated the importance of fostering interparental positivity due to its unique impact on early child behavioral development, it has not necessarily been able to instill a sense of urgency given that child behavioral outcomes are still relatively malleable across the lifespan (Shapiro et al., 2015; Stuebe et al., 2021). Conversely, prior research on the crucial role of direct caregiving behavior as well as intimate partner violence for infant neural development effectively created a sense of urgency, specifically highlighting the need to prioritize the enhancement of positive direct caregiving as well as the prevention of negative direct caregiving and intimate partner violence (Stuebe et al., 2021). As such, the current study results are crucial in underscoring the importance of a comprehensive approach, recognizing the additional unique importance of interparental positivity for neural development during the early sensitive period of infancy. Specifically, intervention initiatives may focus on helping couples navigate the challenges of the early postpartum period, while promoting positive relationship dynamics.

A crucial lingering question pertains to whether particular aspects of infants’ positive vicarious social experiences may be more critical for shaping their developing brains than others. In our current study, observed interparental positivity was operationalized as a single, yet multidimensional, construct. Specifically, our measurement encompassed various characteristics such as engagement, fun, positive affect and sensitivity (Frosch and Mangelsdorf, 2022). It remains plausible that not all these relational components hold equal importance for vicariously shaping infants’ developing brains. Due to the pioneering nature of the current study, it was not designed with sufficient statistical power to conduct in-depth examinations involving multiple comparisons across various facets of vicarious experiences. Future research may be specifically built to address this gap.

Importantly, it should be noted that the current study identified an association between positive aspects of couple interactions and infants’ relative left frontal resting alpha EEG asymmetry, only for observed measures of mother–father interactions, and not for self-reported indices. These results might be interpreted in light of previous research that has consistently indicated disparities between self-reported information and observed measures of human behavior (Bennett et al., 2006; Herbers et al., 2017), as well as growing discrepancies for sensitive constructs such as those related to social interaction (Bennett et al., 2006; Herbers et al., 2017).

In the current study, alongside the previously discussed covariate of interparental negativity, several other important covariates were considered, including caregivers’ direct warm and responsive interactions with the infant, to further examine the unique importance of interparental positivity for infant neural development. Notably, the significance of interparental positivity held above and beyond the impact of all these additionally considered covariates. However, it is important to mention that while some of these covariates demonstrated “practically” significant associations with relative left infant frontal alpha EEG asymmetry (e.g., warm and responsive caregiving behavior father; Ferguson, 2016), others did not (e.g., warm and responsive caregiving behavior mother), and none of these covariates showed statistically significant links. Related to the previously discussed point, the absence of significant associations in the current study for these control variables, as opposed to findings from previous research, could be attributed to the reliance on parent reports, which might be particularly problematic when assessing sensitive constructs such as warm and responsive caregiving for infants (Bennett et al., 2006; Herbers et al., 2017). Specifically, in a societal context that heavily emphasizes the postpartum significance of nurturing infants, parents might find it particularly challenging to self-report on lower levels of this factor (Scharf and Natan, 2022). This challenge may be even larger for mothers, as compared to fathers, since mothers are still generally considered primary caregivers, and therefore may be more exposed to these societal pressures. This may potentially account for the current study’s findings showing a weaker connection between maternal direct caregiving and relative left infant frontal alpha EEG asymmetry compared to paternal direct caregiving. As such, future research should include behavioral coding of both maternal and paternal warmth towards the infant and control for these factors to ensure more accurate results.

Moreover, much like interparental positivity, also direct parental warm and responsive caregiving behavior is a complex and multifaceted construct (Ilyka et al., 2021). Previous research has indicated how various facets of warm and responsive caregiving behavior are associated with infants’ neural response pattern, including frontal resting alpha EEG asymmetry (for review see Ilyka et al., 2021). Yet, insights from past work on both animals (Baram et al., 2012; Dettmer et al., 2016) and human infants (Feldman, 2007; Meltzoff, 2007; Mesman, 2010) highlight the specific significance of one particular dimension, i.e., parents’ contingent responsiveness. Contingent responsiveness entails the microanalytic characteristics of parental warm and responsive caregiving behavior, emphasizing the extent to which parents’ behavior is temporally predicted by and contingent upon infant changing cues within rapid moment-to-moment communications. For example, when comparing two mothers who invest equal amounts of time in caring for their infants, particularly mothers who do so consistently and in response to their infants’ cues tend to contribute to more favorable developmental outcomes (Baram et al., 2012; Dettmer et al., 2016).

Moreover, some of the self-reported measures, particularly those from fathers, showed relatively low Cronbach’s alpha reliability values. A potential explanation is that throughout recruitment mainly mothers showed high motivation to participate. As such, some fathers may have participated in the study primarily to please their partners, potentially affecting their motivation and accuracy while filling out questionnaires. In summary, future research may focus on enhancing the reliability of parents’ self-reported data, as well as incorporate observed data, while also examine individual components of multi-dimensional constructs. Together, these steps will contribute to achieving a more nuanced and comprehensive understanding.

Future work might assess the relevance of coparenting quality (Cowan and Cowan, 1996). Coparenting quality encompasses the specific characteristics of the mother–father relationship that are relevant to raising the child. It includes aspects such as coparenting cooperation (i.e., the extent to which parents support each other’s parenting strategies both emotionally and instrumentally), coparenting competition (i.e., the extent to which parents vie for the child’s attention), coparenting warmth (i.e., the extent to which a partner shows affection for the other partner when they are interacting with the child) and coparenting pleasure (i.e., the degree to which parents take delight in how their partner interacts with their child). It is possible that particularly these coparenting aspects are important sources of children’s vicarious social experiences building their brains, as children take an active part in the interactive context in which they unfold. As such, future research might be specifically built to assess the importance of these characteristics.

Measuring resting state EEG in infancy is a complicated task. Resting state recordings aim to capture the brain’s activity when participants are not engaged in any specific cognitive tasks, reflecting their ‘resting’ or ‘idling’ brain state. In adults, resting state EEG is typically recorded while the participant is asked to sit relaxed and quietly with their eyes closed, while staying awake and minimize eye and body movements. For infants, this procedure is not possible, and no other standard procedures have been established. Therefore, in this study, we adopted a common approach of using machine-blown bubbles and having the infant sit on the lap of a familiar person. This method was intended to help the infant remain relaxed and at rest rather than cognitively occupied, during the potentially stressful experience of wearing the EEG cap. Nevertheless, it is important to acknowledge that this situation could have been cognitively demanding after all, at least for some of the infants, which might have caused bias. For instance, infants who are regularly exposed to positive interactions between their parents might show more favorable neural responses to their mother’s presence. This could potentially explain the observed greater left frontal EEG asymmetry measures. Future research should be designed to test this, as well as other alternative explanations related to the measurement of resting EEG in infants. For instance, studies could explore various resting state scenarios, such as having the child sit in a highchair or on a stranger’s lap, to assess whether these variations influence the study outcomes.

It is crucial to note that the current study was designed to uncover correlational associations between interparental positivity and infant frontal resting alpha EEG asymmetry. As such, no causation or directionality may be discerned, and alternative explanations than those detailed above may explain the current study’s results. For example, infant development may have affected the dynamics of the early postpartum couple relationship. Alternatively, a third variable such as genetic predisposition or shared temperamental characteristics between parent(s) and child may have played a role in shaping both infant development and the couple’s dynamics. To further untangle these options, future research may employ repeated measures over time to assess directionality, as well as employ experimental manipulations or interventions aimed at manipulating interparental positivity to shed light on causality (Shmueli, 2010). Moreover, future research could benefit from larger sample sizes, which would allow for the reliable application of additional statistical tests, such as Bonferroni correction, without significantly increasing the risk of Type II errors (Nakagawa, 2004; Perneger, 1998).

In addition to the above discussed suggestions for further research, various other aspects warrant attention to be addressed in future work. First, the importance of additional constellations of vicarious social experience may be assessed. For example, our study exclusively involved heterosexual couples. While in line with extensive previous work (Suárez et al., 2023), we do not presume differences in caregiving dynamics between different couple configurations, future research might be specifically built to assess potentially differential vicarious social impact. Similarly, the relevance of inter-caregiver relationships beyond parents, such as those among non-parental caregivers in daycare facilities, may be examined. Related, our study attracted exclusively white and predominantly Jewish participants, the majority of which were mid-class or high earners. As such, generalizability of results is limited to families with similar socioeconomic and cultural backgrounds and future research might be particularly designed to increase sample diversity.

Second, future research may incorporate infant socio-emotional developmental outcomes, such as gaze following, imitation, or empathy, in order to validate whether the mother–father relationship shapes the infant brain to an extent that ultimately contributes to these social child outcomes that can be reliably assessed at early age (Brooks and Meltzoff, 2005; Roth-Hanania et al., 2011). While such a connection is strongly anticipated based on our study’s findings indicating robust links between the mother–father relationship quality and the infant brain, as well as previous research delineating strong associations between infant and young children’s frontal alpha EEG asymmetry and behavioral social outcomes (Frenkel et al., 2024; Fox et al., 1995; Mundy et al., 2000; Paulus et al., 2013), it remains essential for future research to rigorously examine the full mediation model. Such comprehensive approach will strengthen the current study’s initial evidence for a vicarious experience-brain-behavior pathway.

Third, all constructs relevant for the current study may be validly measured at ages preceding 12 months (Brooks and Meltzoff, 2005; Marshall et al., 2002; Roth-Hanania et al., 2011). Moreover, developmental neuroscience has hinted towards the occurrence of vicarious learning as early as 7–8 months (Addabbo et al., 2020; Drew et al., 2018; Missana and Grossmann, 2015). As such, future work may include repeated measures over time, not only to allow for the previously mentioned assessment of directions of effects but also to enable the unraveling of the specific age at which vicarious social experiences may begin to shape the infant social brain.

The current study boasts several strengths, among which the inclusion of both mothers and fathers. In addition, infant EEG research is known to be a particularly challenging endeavor, resulting in a typical loss of approximately 25% of participants due to infant unsettledness and technical difficulties (Van der Velde and Junge, 2020). To overcome these challenges, the current study greatly invested in a three-headed research team, featuring two mid-adult research assistants with over 15 years of experience working with infants and parents. Additionally, a more junior research assistant, though not directly involved in interactions with infants or parents, provided valuable support to the more experienced assistants. Aside from the probably beneficial effect of a concise home visit, this meticulously assembled research team achieved a minimal loss of 13% of infants due to EEG data collection interferences. The latter significantly contributed to minimizing bias and enhancing the overall robustness of data (Van der Velde and Junge, 2020).

### Practical recommendations

The findings from the current study serve as a pioneering step in recognizing the vital significance of positive postpartum couple relationships in shaping infants’ neural substrates of adaptive social development. The current study’s insights provide a compelling opportunity for highlighting the importance of the development and implementation of prevention and intervention initiatives targeting new parents. Specifically, these initiatives may aim to nurture and enhance the mother–father relationship by providing tailored support and resources, both through existing post-partum initiatives as well as programs specifically designed to address couples’ needs.

## Data Availability

The raw data supporting the conclusions of this article will be made available by the authors, without undue reservation.
